# Evaluating the clinical efficacy of pulsed dye laser with sirolimus for treatment of capillary malformations: A systematic review

**DOI:** 10.1002/ski2.333

**Published:** 2024-01-07

**Authors:** Isabella J. Tan, Thu M. Truong, Gaurav N. Pathak, Shaunt Mehdikhani, Babar Rao, Bernard A. Cohen

**Affiliations:** ^1^ Robert Wood Johnson Medical School, Rutgers The State University of New Jersey Piscataway New Jersey USA; ^2^ Department of Dermatology The Johns Hopkins Hospital Baltimore Maryland USA

## Abstract

Port‐wine stains (PWS) are capillary vascular anomalies that are often treated with pulsed‐dye laser (PDL). Revascularization limits persistent clearance; however, the anti‐angiogenic effects of sirolimus (SIRO) may inhibit revascularization. This review aims to determine differences in PWS outcomes when treated with PDL monotherapy or in combination with SIRO. A systematic review was conducted using PubMed, Cochrane, and Embase databases. The following search terms were used: ‘port wine stain PDL SIRO’, ‘port wine stain PDL’, and ‘port wine stain PDL and topical treatment’ with (MeSH) and (Title/Abstract) limits. The search was limited to the English language and human‐subject studies conducted between 1 January 2000 and 1 June 2023. Inclusion criteria included studies evaluating SIRO as an adjunct to PDL in patients with PWS. Data extraction and quality assessment were performed by two independent reviewers. A total of nine studies met the inclusion criteria, which included randomized controlled trials (3), case series (2), case reports (3), and a prospective intrapatient study (1), which represented a total of 58 patients. Five studies showed improvement of a measured post‐treatment PDL parameter including shortening treatment time and less frequent dosing. A subset of studies (4/9) which did not demonstrate significant clinical improvements exhibited significant photographic evidence of improvement. Heterogeneity among the studies highlights the need for further research and standardization. While adjunctive SIRO shows promise, larger studies and comprehensive evaluation methods are required to establish conclusive safety and efficacy guidelines to shape clinical decision‐making.

## Graphical abstract

This systematic review examines the use of sirolimus in combination with pulsed‐dye laser (PDL) therapy for treating port‐wine stains (PWS), a type of vascular anomaly. The review includes nine studies involving a total of 58 patients and finds that while some studies reported improvements in PWS treatment outcomes, there was heterogeneity amongst the results. Further research and standardized evaluation methods are needed to establish clear guidelines for the safety and efficacy of combining sirolimus with PDL in treating PWS, despite its potential promise.

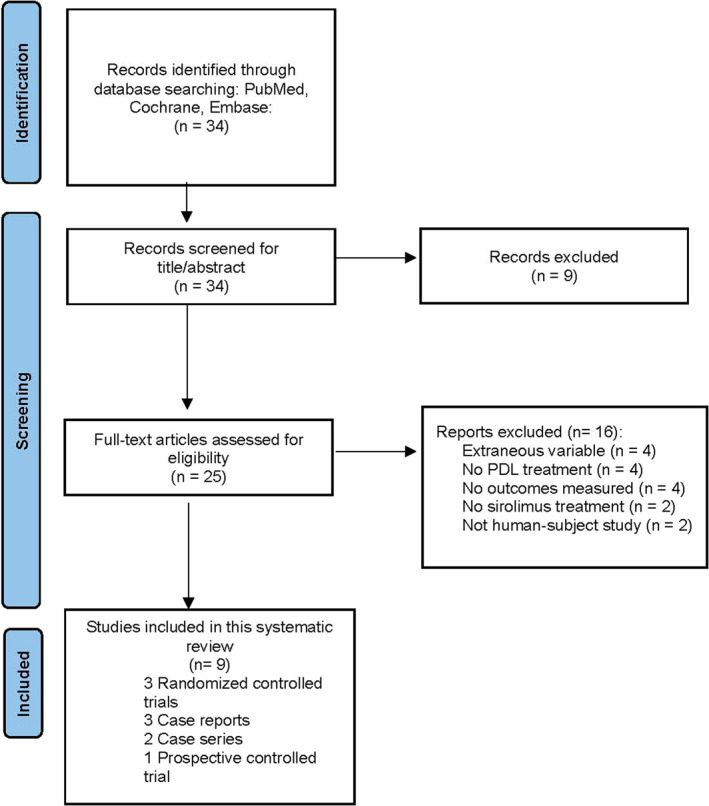

**What is already known about this topic?**

- Port‐wine stains (PWS) are vascular anomalies often treated with pulsed‐dye laser (PDL). Sirolimus (SIRO), with potential anti‐angiogenic effects, is explored as an adjunct to PDL. A systematic review of nine studies found mixed results, with some indicating improved PDL outcomes when combined with SIRO, including shorter treatment times and less frequent dosing. However, heterogeneity among studies underscores the need for more extensive research and standardized evaluation methods to establish safety and efficacy guidelines for clinical decision‐making.

**What does this study add?**

- This study adds to the existing knowledge by systematically reviewing the use of SIRO as an adjunct to PDL for treating PWS. It highlights mixed results from various studies, indicating potential benefits in terms of improved PDL outcomes. However, it also emphasizes the need for further research and standardization due to the heterogeneity among the included studies. Ultimately, this study underscores the importance of continued investigation to establish conclusive safety and efficacy guidelines for the use of SIRO in PWS treatment.

## 1 INTRODUCTION

The mainstay of therapy for the treatment of port‐wine stains (PWS) are vascular‐selective lasers, such as pulsed‐dye laser (PDL). PDL treatment is limited by the need for multiple sessions, and that complete and lasting blanching is rarely achieved with monotherapy.^1^, ^2^ Additionally, there is significant variance in individual treatment response, as 20%–46% of patients are partial responders (20%–30% with PDL) and 14%–40% of patients are poor responders, despite multiple treatments.^3^, ^4^ PWS therapeutic approaches have not changed longitudinally since the late 1980s, indicating an unmet medical need despite present‐day advances in both technological and pharmacologic interventions.^5^

Neovascularization post‐PDL treatment may result in recurrence or suboptimal response. To improve PDL success, topical agents with antiproliferative properties have been evaluated as adjunctive therapies to PDL, in hopes of improving longitudinal clinical effects.^6^ Sirolimus is a cell‐cycle‐specific mammalian target of rapamycin (mTOR) inhibitor that blocks cell‐cycle progression, thereby causing immunosuppressive and antiproliferative effects.^7^ Dual PDL and topical SIRO therapy have been reported to improve PWS clearance over a shorter duration of time and with fewer PDL treatments.^6^, ^8^ However, despite these advancements, challenges persist in achieving complete clearance due to factors such as vessel depth variability, tissue characteristics, and individual response variations.^5^ This systematic review aims to determine if there is a significant difference in utilizing adjunctive topical SIRO in this indication while addressing its implications for the future treatment of PWS.

## 2 METHODS

A systematic literature review was conducted using PubMed, Cochrane, and Embase databases to assess the role of topical SIRO therapy on PDL treatment outcomes for PWS. The review was conducted in accordance with PRISMA guidelines and the following search terms were used: ‘port wine stain PDL SIRO’, ‘port wine stain PDL’, and ‘port wine stain PDL and topical treatment’. The search was limited to studies conducted between 1 January 2000 and 1 June 2023. Inclusion criteria included all study types in English, and those that evaluated the use of SIRO with PDL. Exclusion criteria was set as papers in a language other than English, not including humans as subjects, or not evaluating SIRO as the adjunct to PDL.

**FIGURE 1:**
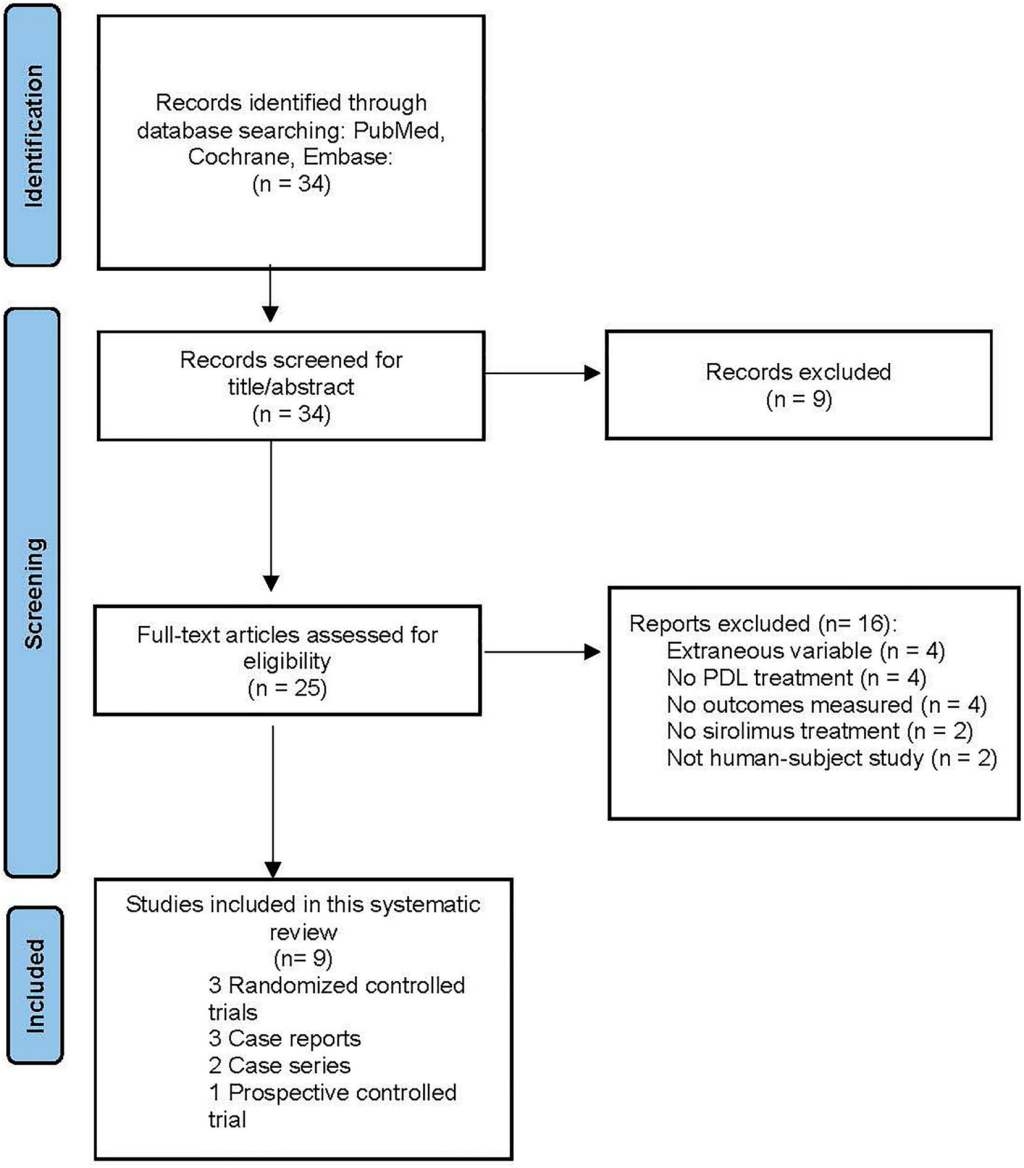
Flowchart illustrating the article inclusion process.

A total of 34 records were identified from the initial search (Figure 1). After exclusion, a total of nine studies were included in the analysis and data abstraction.

Two reviewers (I.J.T. and T.M.T.) independently extracted the data, with disagreements resolved by consensus. To determine study quality, two authors (I.J.T. and T.M.T.) independently used a previously validated 5‐point scale with values of 0 or 1, including assessment of exposure and outcome, control of confounding factors, and evaluation of bias.^9^ Scores were summed and those ranging from 0 to 3 were regarded as lower in quality, and scores of 4–5 were considered higher quality. The certainty of evidence table was generated using GRADEpro (Supplemental 1).^10^

During data abstraction, there was considerable variability in study characteristics to yield a meaningful summary of the estimate of effect, thus the included studies were analyzed according to synthesis without‐meta‐analysis (SWiM) guidelines (Supplemental 2).^11^ Thematic synthesis was used to harmonize findings in qualitative research.

## 3 RESULTS

### 3.1 Study selection

A total of nine studies were assessed for the clinical outcomes of adjunctive SIRO and PDL for treatment of capillary vascular malformations. Studies that appeared to meet inclusion criteria included animal and cell‐based models of capillary malformations treated with SIRO and PDL; however, these were later excluded based on exclusion criteria.^12^, ^13^ The study characteristics and study population characteristics are outlined in Table 1.

**TABLE 1:** Study characteristics and clinical characteristics.

Study	Study design	Patient characteristics				Evaluation intervals	Treatment arm	Control arm	Total # of cases/completed study
		Location (*n*)	Photo‐type (*n*)	% female	Mean age (*y*)				
Fallahi et al., 2021	DBP split lesion RCT	Centrofacial (with sub‐analysis of lateral vs. medial) or neck (15)	I (1), II (2), III (8), IV (4)	46.7	21.76	Baseline, 2 months, 4 months, 6 months, and 8 months after baseline	Full stain: PDL	Full stain: PDL	20/15
							Half stain: SIRO 0.2% cream applied once nightly after each PDL session for 8 months	Half stain: Vehicle cream applied nightly after each PDL session for 8 months	
Greveling et al., 2017	Prospective, intra‐patient RCT	Extrafacial, neck, trunk or extremities (14)	II (6), III (8)	71	31	1, 3, 6, and 12 months after last intervention	Three of four 1‐cm^2^ squares within stain: PDL followed by SIRO 0.1% solution left in situ for 7 days; OR PDL followed by Er:YAG and SIRO 0.1% solution left in situ for 7 days; OR SIRO 0.1% solution left in situ for 7 days	One of four 1‐cm^2^ squares within stain: PDL monotherapy	17/14
Griffin et al., 2016	Case report	Centrofacial, neck, chest (1)	Not reported	0	55	Baseline, after initial treatment, and after follow‐up treatment	Initial: 11 sessions PDL monotherapy	N/A	1/1
							Follow‐up: 4 sessions PDL followed by SIRO 0.5% ointment twice daily		
Marqués et al., 2015	RCT	Centrofacial, neck (23)	II and III (23)	52	33	6, 12, and 18 weeks after the intervention	Each of three quarter stains: PDL followed by vehicle cream daily for 12 weeks; OR PDL followed by SIRO 1% cream daily for 12 weeks; OR SIRO 1% cream daily for 12 weeks	One quarter stain: Topical vehicle daily for 12 weeks	23/12
Musalem et al., 2018	Case series	Centrofacial (2), extremities, chest (3)	Not reported	60	11	Not reported	PDL monotherapy followed and SIRO 1% (or SIRO 0.5%) cream daily; ranging from 4 to 11 months of therapy	N/A	5/5
Nelson et al., 2011	Case report	Chest (1)	I and II (1)	0	37	Every 2–4 weeks for 13 months	Three of six 3‐cm^2^ test sites within stain: SIRO 2‐mg tablet orally once daily to establish steady‐state concentration followed by PDL and continual SIRO 2‐mg tablet orally once daily for 4 weeks	Three of six 3‐cm^2^ test sites within stain: PDL only	1/1
Junco et al., 2019	Case report	Centrofacial, laterolfacial and scalp (1)	I (1)	100	69	At birth, 3 weeks, 10 months, 23 months	Systemic SIRO 0.25 mg/12 h (0.8 mg/m^2^/12 h), and systemic aspirin at 10 mg/kg/d at 3 weeks of age, followed by PDL at 2 months of age	N/A	1/1
Artzi et al., 2019	Case series	Left arm (1), laterofacial (2)	II (2), III(1)	66	12.67	Baseline, before PDL treatment, 4 weeks after last PDL treatment	Half stain: PDL followed by tixel up to 14 days after with SIRO 0.2% cream immediately, and twice daily after	Half stain: PDL followed by SIRO 0.2% cream twice daily	3/3
Doh et al., 2017	Open‐label, prospective intrapatient	Trunk (4), extremities (2)	III and IV (6)	100	31.5	Baseline, 8 and 16 weeks after baseline	Two of three 1‐cm diameter circular sections of stain: PDL followed by SIRO 1% cream once daily for 1 week after first PDL session; OR PDL followed by SIRO 1% cream once daily for 8 weeks after first PDL session	One of three 1‐cm diameter circular sections of stain: PDL followed by vehicle cream once daily for 8 weeks after first PDL session	6/6

Abbreviations: DBP, double‐blind placebo‐controlled; Er:YAG, Erbium YAG laser; PDL, pulsed dye laser; RCT, randomized controlled trial; SIRO, sirolimus.

Overall, a majority (5/9) of the included studies were of high quality based on the evaluation of study design, assessment of exposure, assessment of outcome, control for confounding, and evidence of bias. The certainty of evidence evaluation showed that for the outcome of ‘colourimetric improvement’ in three studies reflected moderate certainty, whereas the outcome of ‘subjective clearance’ assessed by photographic evaluation showed low certainty, attributed to a high risk of publication bias and inconsistency in the evaluation of methods.

Across all nine included studies, there were a total of 75 patients. There were no statistically significant differences in the three studies that utilized colourimetric analysis.^14^, ^15^, ^16^ However, seven of the studies reporting subjective clearance by photo evaluation reported a statistically significant improvement.^16^ In all studies evaluating patient satisfaction, two studies identified that despite no measurable colourimetric improvement, both had improved patient satisfaction scores.^15^, ^16^ This analysis is summarized in Table 2.

**TABLE 2:** Clinical outcomes of combined sirolimus and PDL therapy.

Study	PDL characteristics		Sirolimus characteristics		Method of clinical outcome evaluation	Clinical outcomes	Summary	Limitations
	No. of treatments	Brand; fluence (J/cm^2^); pulse duration (ms)	Topical strength	Preparation				
Doh et al., 2017	2–3	VBeam perfecta; 4.75–6.25; 0.45	1.00% w/v	300 2‐mg SIRO tablets crushed and mixed in 12% ammonium lactate lotion	Chromameter	Vascular erythema; colour difference; blanching rate	Combined topical SIRO at 1.00% concentration and PDL therapy for PWS showed no significant difference in erythema, colour difference, or blanching rate between PDL monotherapy and combined sirolimus regimens	Open labelled pilot study; non‐randomized; small sample size
Fallahi et al., 2021	4	VBeam perfecta; 12; 1.5	0.20% w/v	1‐mg SIRO tablets ground and mixed with propylene glycol (2.5%), polysorbate (2.5%), and Farabi® base cream	Colourimetric (visioface)	Colourimetric analysis	PDL and topical SIRO at 0.20% concentration showed no significant difference in colourimetry between placebo and sirolimus groups, but subjective assessments (IGA and PGA scores) indicated significant improvement in the treatment group	Loss to follow‐up; small sample size
Greveling et al., 2017	3	VBeam perfecta; 7–11; 0.5–2	0.1% w/v	Not reported	Colourimetric assessment, photo evaluation, patient satisfaction, treatment related pain	Vancouver scar scale; patient and observer scar assessment scale	PDL and topical SIRO at 0.1% concentration showed no significant differences in colourimetric assessment between PDL monotherapy and experimental treatments. Photo evaluation showed a significant difference between PDL monotherapy and SIRO monotherapy, and patient satisfaction was highest with PDL monotherapy, followed by various combination therapies and SIRO monotherapy	Early termination of patient inclusion; exclusion of facial lesions
Griffin et al., 2016	11	VBeam perfecta; 9–11; 0.45–1.5	0.5% w/v	Not reported	Subjective cosmetic improvement and lesion blanching	Cosmetic improvement; nodularity; colour difference	PDL and topical SIRO at 0.5% concentration showed greater cosmetic improvement in appearance, nodularity, and colour of PWS compared to PDL monotherapy after 11 treatments. Follow‐up at 21 months showed persistent blanching of the lesions	Single subject report; control comparison limited
Marqués et al., 2015	1–3	VBeam perfecta; 8–10; 1.5–10	0.10% w/v	1% SIRO powder dissolved in 3.8% benzyl alcohol solution	Blinded photograph evaluation by 2 independent dermatologists	Scale improvement score; digital photographic image score; percentage of vessels in histologic analysis; treatment tolerability	Topical SIRO at 0.1% concentration with PDL was found to be a safe and effective treatment for PWS, as evidenced by improvements in various assessment measures such as scale improvement score, digital photographic image score, percentage of vessels in histologic analysis, and tolerability	PDL applied only to peripheral areas of PWS; older patient population; No long term follow‐up evaluation
Musalem et al., 2018	Varied: 22, 8, 14, 9, 17	Not reported; 7–12; 0.5	0.5%–1% w/v	Not reported	Subjective clearance	Colour change; texture improvement; reduction in thickness; patient satisfaction	Topical SIRO at 0.5%–1% concentration demonstrated an effective response in resistant PWS, leading to subjective clearance characterized by colour change, texture improvement, reduction in thickness, and increased patient satisfaction.	Control comparison limited
Nelson et al., 2011	Not reported	Not reported; 7, 8, 10; 1.5	2 mg/day (oral)	Not reported	Subjective clearance	Blanching response	Oral SIRO at 2 mg/day shows potential feasibility in improving the blanching response of PWS, although topical formulation may be necessary to minimize risk of side effects	Single subject report; control comparison limited
Junco et al., 2019	10	VBeam perfecta; 8–10; 0.45–1.5	0.25 mg/12 h (oral)	Not reported	Subjective clearance	Colour difference	Oral SIRO at 0.25 mg/12 h demonstrated subjective clearance and lightening of PWS during a 23‐month follow‐up period	Single subject report; control comparison limited
Artzi et al., 2019	2–3	Cynergy, cyanosure; 8.5–10; 0.45–1.5	2% w/v	Not reported	Subjective clearance: four blinded dermatologists	Photographic evaluation from baseline (5‐point scale) by patients and physicians; patient satisfaction and tolerability	Addition of tixel with topical SIRO at 2% concentration after PDL treatment resulted in improved subjective clearance rated by both physicians and patients. Patients reported increased satisfaction and adequate tolerability	Small sample size; older patient population

Abbreviations: IGA, investigator's global assessment; PDL, pulsed dye laser; PGA, physician's global assessment; RCT, randomized controlled trial; SIRO, sirolimus.

### 3.2 Colourimetric analysis

Doh et al. conducted an open‐label, prospective intrapatient study comparing the effects of PDL monotherapy against adjunctive topical SIRO regimens.^14^ However, no statistically significant difference was found in erythema, colour difference, and blanching rate between the two groups. The study involved six cases, with treatment arms consisting of either 1 week or 8 weeks of topical SIRO after the first PDL session, while the control arm received PDL monotherapy with two sessions spaced 8 weeks apart.^14^ Greveling et al., conducted an intra‐patient randomized controlled trial (RCT) with 17 cases, but did not find evidence supporting the efficacy of adjuvant SIRO in improving PWS blanching based on colourimetric analysis, despite improved patient satisfaction scores.^16^ Similarly, Fallahi et al. conducted a double‐blind, placebo‐controlled, split‐lesion RCT involving 20 cases.^15^ The results showed no significant difference in colourimetry between the placebo and SIRO groups.^15^

### 3.3 Subjective clearance

Subjective clearance of PWS was improved in five studies, including an RCT^9^ and multiple case series.^6^, ^8^, ^17^, ^18^, ^19^ Particularly for treatment‐resistant PWS and other capillary malformations, SIRO was found to improve PWS clinically.^6^, ^19^ In addition, the use of topical SIRO may be a feasible approach to reduce the risk of systemic side effects.^17^

## 4 DISCUSSION

The results are consistent with the literature that dual therapy with PDL and SIRO has mixed clinical efficacy. Studies evaluating patient satisfaction reflected subjective improvement, despite a lack of statistically significant colourimetric difference. Colourimetric analysis has been utilized as a measure of treatment efficacy in studies examining PWS and other vascular lesions. However, while colourimetry provides an objective quantification of colour changes, it may not capture the full complexity of PWS lesions, which can involve variations in vessel density, depth, and distribution.^20^ Furthermore, colourimetry primarily focuses on the superficial layers of the skin and may not reflect changes in deeper vascular structures.^20^ This limitation may be relevant in cases where significant changes occur at deeper cutaneous levels following treatment.^21^ Additionally, colourimetry may not adequately capture subjective aspects, such as texture or thickness of the lesion. It is essential to consider that PWS and other vascular lesions encompass diverse characteristics, making a comprehensive assessment challenging through colourimetric analysis alone.^22^ Therefore, while colourimetry can provide valuable quantitative data, its limitations must be acknowledged and a comprehensive evaluation incorporating other clinical and subjective measures should be employed to effectively assess treatment outcomes.

### 4.1 Facial PWS

Five studies involved centrofacial sites.^6^, ^8^, ^9^, ^15^, ^18^ The largest study by Fallahi et al. examined centrofacial PWS lesions, with a sub‐analysis of lateral and medial lesions. They reported no significant difference in facial PWS colourimetry between the placebo and SIRO groups, however, subjective assessments showed greater improvement in the SIRO treatment group.^15^ Similarly another RCT by Marqués et al. showed topical SIRO combined with PDL being a safe and effective treatment for capillary vascular malformations.^9^ However, the study mainly focused on adult subjects and laterofacial sites were also used as control areas which may be subject to treatment bias as previous studies show these sites are more difficult to treat. Additionally, smaller case studies that included centrofacial areas noted improvement.^6^, ^8^, ^18^

### 4.2 Extrafacial PWS

Doh et al. found no significant difference in erythema, colour difference, or blanching rate between PDL alone and combined topical SIRO regimens.^14^ This suggests the limited efficacy of the combination therapy for extrafacial PWS, which is often characterized by deeper vessels. Greveling et al., observed no statistically significant difference in colourimetric assessment between PDL monotherapy and combined topical SIRO regimens. However, patient satisfaction was highest with PDL monotherapy.^16^ Thus, these findings indicate that PDL alone may be more effective than combined SIRO and PDL for extrafacial PWS.

### 4.3 Effect of variation in sirolimus formulations

Seven of the nine included studies evaluated a variety of topical SIRO formulations ranging from 0.1% to 2% in weight/volume proportions, which at some doses may require compounding in the pharmacy. Two studies evaluated oral SIRO in 0.5–2 mg daily dose ranges. A lack of standardization in dosing strength and formulation type may make intra‐study comparisons challenging and potentially account for differences in the observed clinical outcomes.

Topical SIRO has minimal systemic absorption. Three of the seven studies utilized topical SIRO with similar or lower concentrations as compared to commercially available SIRO 0.2%, while the remaining four studies utilized higher topical concentrations. The varying concentrations may contribute to differences in statistically significant clinical efficacy, although all studies revealed improved subjective clearance. Artzi et al., used a 0.5%–1% SIRO cream and PDL combined with a non‐laser skin resurfacing system (Tixel) to overcome limitations of low drug bioavailability. Therefore, other permeation‐enhancing techniques may be a suitable option for deeper or more hypertrophic‐type PWS.

### 4.4 Tolerability of PDL and sirolimus

PDL can induce temporary skin inflammation and compromise the skin barrier, which may enhance the irritant effects of topical SIRO. Although the topical formulation is more favourable for targeted delivery, appropriate dosage must be used to manage the potential adverse effects of SIRO, which include skin dryness and contact dermatitis.

Patients with Fitzpatrick phototypes IV–VI are more susceptible to adverse events when undergoing PDL treatment for PWS compared to patients with other skin types.^23^ While PDL has shown improvements in PWS in individuals with skin of colour, there is risk of hyperpigmentation, hypopigmentation, and scarring that needs to be carefully considered.^23^ The potential of SIRO to reduce the number of treatment sessions required for PWS blanching may be particularly advantageous for patients with skin of colour, as it may minimize the risk of adverse effects associated with prolonged or repeated PDL treatments.

### 4.5 Limitations

The limitations of the reviewed studies include several factors that may impact the interpretation and generalizability of the findings. First, a small number of studies met the inclusion criteria and most studies had small sample sizes, with seven out of nine studies having less than 10 participants. This reduces the statistical power and limits the ability to draw significant conclusions.

Another consideration is the age distribution of the participants. Although PWS are commonly observed in children, the average age of the participants across the studies was relatively higher. Five out of the nine studies had participants in their 3rd decade of life, and two studies included participants in their 5th to 6th decade of life. This age discrepancy raises questions about the potential impact of age on treatment outcomes, specifically whether older individuals may exhibit decreased angiogenesis that can significantly impact treatment response and outcome measures.^24^ Anatomic differences also pose limitations in the interpretation of the findings. Centrofacial lesions tend to respond less favourably to treatment compared to lesions in other locations.

Additionally, the cost of compounding high SIRO dosages is a significant limitation in the reviewed studies. This cost factor should be taken into consideration when evaluating the practicality and feasibility of adjunctive SIRO therapy. The lack of standardization in the formulation type and dosage may make inter‐study comparisons challenging. Exploring alternative delivery vehicles may offer a potential avenue to reduce compounding costs and enhance treatment outcomes.

While this review suggests that adjunctive SIRO with PDL therapy may subjectively improve PWS outcomes, factors such as age, location of the PWS, and SIRO concentration should be appraized to guide clinician recommendations. Additional research exploring alternative drug delivery systems and treatment modalities is warranted to enhance the safety and efficacy of PWS therapies.

## 5 CONCLUSION

This systematic review identified mixed efficacy of adjunctive SIRO in the treatment of PWS with PDL. Subjective clearance assessed through photographic evaluation consistently demonstrated improved patient satisfaction, however, statistical analysis frequently exhibited no significant difference. Further research with standardized protocols and larger patient cohorts is necessary to fully understand the safety and efficacy of SIRO and PDL treatment for PWS.

## AUTHOR CONTRIBUTIONS

**Isabella J. Tan**: Conceptualization (lead); data curation (lead); formal analysis (lead); funding acquisition (lead); investigation (lead); methodology (lead); project administration (lead); resources (lead); software (lead); validation (lead); visualization (lead); writing – original draft (lead); writing – review & editing (lead). **Thu M. Truong**: Conceptualization (lead); data curation (lead); formal analysis (lead); funding acquisition (lead); investigation (lead); methodology (lead); project administration (lead); resources (lead); software (lead); validation (lead); visualization (lead); writing – original draft (lead); writing – review & editing (lead). **Gaurav N. Pathak**: Conceptualization (equal); data curation (equal); formal analysis (equal); funding acquisition (equal); investigation (equal); methodology (equal); project administration (equal); resources (equal); software (equal); validation (equal); visualization (equal); writing – original draft (equal); writing – review & editing (equal). **Shaunt Mehdikhani**: Conceptualization (equal); data curation (equal); formal analysis (equal); funding acquisition (equal); investigation (equal); methodology (equal); project administration (equal); resources (equal); software (equal); validation (equal); visualization (equal); writing – original draft (equal); writing – review & editing (equal). **Barbar Rao**: Supervision (lead). **Bernard A. Cohen**: Supervision (lead).

## CONFLICT OF INTEREST STATEMENT

Dr. Rao is a speaker for Incyte. All other authors have no disclosures.

## ETHICS STATEMENT

Not applicable.

## Data Availability

The data that support the findings of this study are available from the corresponding author upon reasonable request.
